# Three-Level Distributed Real-Time Monitoring of Construction near Underground Infrastructure Using a Combined Intelligent Method

**DOI:** 10.3390/s22093260

**Published:** 2022-04-24

**Authors:** Biao Zhou, Yingbin Gui, Xiaojian Wang, Xiongyao Xie

**Affiliations:** 1 Key Laboratory of Geotechnical & Underground Engineering, Ministry of Education, Tongji University, Shanghai 200092, China; 1932597@tongji.edu.cn (Y.G.); seanwxj@tongji.edu.cn (X.W.); xiexiongyao@tongji.edu.cn (X.X.); 2 Department of Geotechnical Engineering, Tongji University, Shanghai 200092, China

**Keywords:** underground infrastructure, construction monitoring, real-time, mutual information, blind source separation

## Abstract

With the rapid development of underground infrastructure and the uncertainty of its location, the possibility of damage due to nearby construction has increased. Thus, for the early warning of dangerous construction behaviors around underground facilities, this paper proposes a novel real-time distributed monitoring method with three levels, comprised of the terminal node, relay node, and server. Corresponding to these three monitoring levels, a vibration-based intelligent solution for recognizing the construction source is presented and compared with the traditional method. First, the blind source separation method was used to separate collected signals into a limited number of monitoring object sources; this helped to minimize the number of required classification categories and reduce the recognition uncertainty caused by signal mixing. Then, the mutual information (MI) method was used to select suitable vibration features, which were used as the input matrix for the resulting intelligent recognition. Finally, the construction behaviors were identified at the server based on returned features. Guided by this method, a sample dataset including pile-driving, train-operation, and environment-vibration signals was constructed and combined with a multi-layer perceptron (MLP) and a long short-term memory (LSTM) network. The effects of blind source separation and the MI method are discussed in depth in this paper.

## 1. Introduction

As the density of subway and underground pipe networks increases with the rapid development of urban construction, the possibility of damage to these infrastructures owing to nearby construction also increases. Pile foundation construction is among the most common construction behaviors that can affect underground infrastructure. In addition, the uncertainty of the location of existing infrastructures and the randomness of construction behavior has caused several accidents in the past. Indeed, there have been several incidents in the Chinese cities of Chengdu, Shenzhen, and Shanghai, where subway tunnel structural damage has occurred as a result of nearby pile construction, including a pile head hitting an operating train, causing permanent tunnel damage. Damage to underground pipelines is much more common and has resulted in gas pipe explosions in Qingdao, China. Therefore, the development of a real-time monitoring system for nearby construction is of great significance to prevent the destruction of important underground facilities.

However, this is a considerably challenging task. First, most underground facilities have become increasingly networked. For instance, subway tunnels are extremely long linear structures, with the length of a single line typically exceeding 10 km; these lines also pass through different areas. Hence, given the limited monitoring points, realizing early warnings for the damage caused by pile driving and other surrounding construction behaviors is remarkably difficult. In the past, underground infrastructure monitoring has been primarily concerned with structural deformation or leakage, and related settlement monitoring systems have been developed using the static level or optical fiber test methods [1,2]. However, these methods can only cover significantly small areas, and it is difficult to conduct real-time monitoring and realize early warnings for nearby constructions. As construction behaviors, such as pile driving, result in structural soil and tunnel vibrations, a number of vibration or sound source perception and detection methods have been widely applied with seismic [3], noise [4], and acoustic emission (AE) [5] applications; the vibration detection approach can provide the real-time monitoring of nearby construction over a large area.

Second, apart from the vibrations caused by nearby construction, underground spaces comprise various other vibration sources; these mainly include train-induced vibrations and various environmental noises. The interaction and superposition of such vibration signals can generate new recognition categories, which, in turn, can considerably increase the difficulty in recognition. Furthermore, most previously proposed sound- and vibration-based monitoring methods are dependent on factors such as wave propagation properties [6,7] and modal characteristics [8,9,10]. However, in-depth analyses of such characteristics require complex and time-consuming calculations such as waveform-based seismic location methods [11] including full waveform inversion, which depends on complex inversion or reverse imaging algorithms and requires powerful computing resources. Furthermore, previous studies have shown that vibration responses and features are characterized by strong nonlinearity and uncertainty due to the structure and soil interference, especially when applying vibration-based methods in underground infrastructures [12,13]. The aforementioned characteristics and features may change depending on the variations in train speeds, soil properties, tunnel structures, and construction locations; hence, it is difficult to determine specific evaluation ranges for automatic judgments without human intervention. As a result, traditional signal processing methods are unable to directly conduct continuous and stable information perception and online monitoring.

Recently, a number of deep learning networks have been rapidly developed and applied in medical and mechanical fields, such as disease and human activity pattern recognition [14,15,16], and optical and medical signal diagnosis [17]. Deep learning networks have also been employed in applications closer to the focus of this study, such as bridge monitoring data analysis [18], truck loading detection [19], indoor fall detection for the elderly [20], and construction monitoring using sound classification [21]. However, these methods have all encountered various challenges, including the complexities of potential classification types and the need for large sample sets. Considering the difficulties in sample collection and the cases involving signal mixtures, it is necessary to develop new models or employ additional algorithms.

Meanwhile, rapid progress in blind source separation methods [22,23,24,25] has made it possible to develop more efficient models for use in vibration signal classification and recognition. Among these methods, mutual information (MI) analysis can be used to determine the degree of uncertainty associated with the assigned classification category by using several quantified features, such as information entropy [26]. Indeed, Shannon’s MI-based theories [27] have been widely employed in applications such as the selection of multi-channel electroencephalogram features [28]. Furthermore, the combination of MI-based methods and intelligent algorithms has been demonstrated to extend the depth and breadth of feature mining; this approach has achieved continuous progress in recognition accuracy [29,30].

Therefore, considering the challenges associated with intelligent recognition owing to vibration mixing and the difficulties in signal acquisition, a novel real-time monitoring method is proposed. The blind source separation, MI analysis, and deep learning algorithms were combined to develop a limited-sample driven vibration perception and nearby construction classification method. This method can achieve real-time monitoring and early warnings for surrounding construction near underground facilities. Notably, the use of vibration recognition differs from the use of sound recognition in that the former can also reflect the physical characteristics of underground media, and as such, it can be extended in the future to monitor excavations and other construction behaviors, in addition to pile driving.

## 2. Proposed Vibration Recognition Method

### 2.1. Outline of Proposed Method

**Figure 1:**
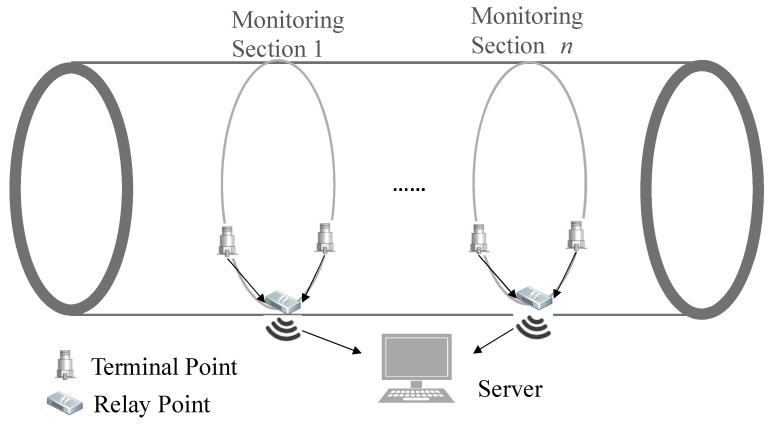
Vibration-based real-time monitoring system for construction behaviors surrounding super-long tunnel structures.

As pile foundation construction is the most common construction behavior affecting underground infrastructure, this study investigated the vibration monitoring method for pile foundation construction near an operating subway tunnel. As shown in Figure 1, based on the distributed layout principle, a novel real-time monitoring method is proposed for the perception of surrounding construction behaviors and early warnings near super-long tunnel structures. This approach mainly consists of three levels, as follows:

- Terminal node for data acquisition and processing

To establish a sensor network covering the entire tunnel network, a super-long subway tunnel is divided into several monitoring sections. At each section, double sensors are symmetrically arranged along the tunnel structure; the section intervals are determined according to the sensing range of the acceleration sensors. For pile-driving vibration monitoring, this interval is typically 200 m; therefore, for a 1 km-long tunnel section, four or five test sections are sufficient. Accordingly, the total number of sensors can be less than ten by arranging two sensors at each section. If necessary, a processing module can be installed on the terminal node for signal sampling and signal processing, in order to reduce data transmission.

- Relay node for mutual analysis and feature screening

In each test section, a relay node is arranged as a terminal server; the connection with the terminal node can be wired or wireless. Moreover, the MI of the sampled signals between terminal nodes is further explored; time or frequency features will be processed here to meet the demands of source recognition. This will also further reduce the data transmission from the relay node to the server.

- Intelligent recognition of construction behavior at server

Finally, the processed data at the relay node are sent to the server via wireless transmission or optical fibers; the intelligent algorithm is then used to identify construction behaviors and provide warnings, if required.

**Figure 2:**
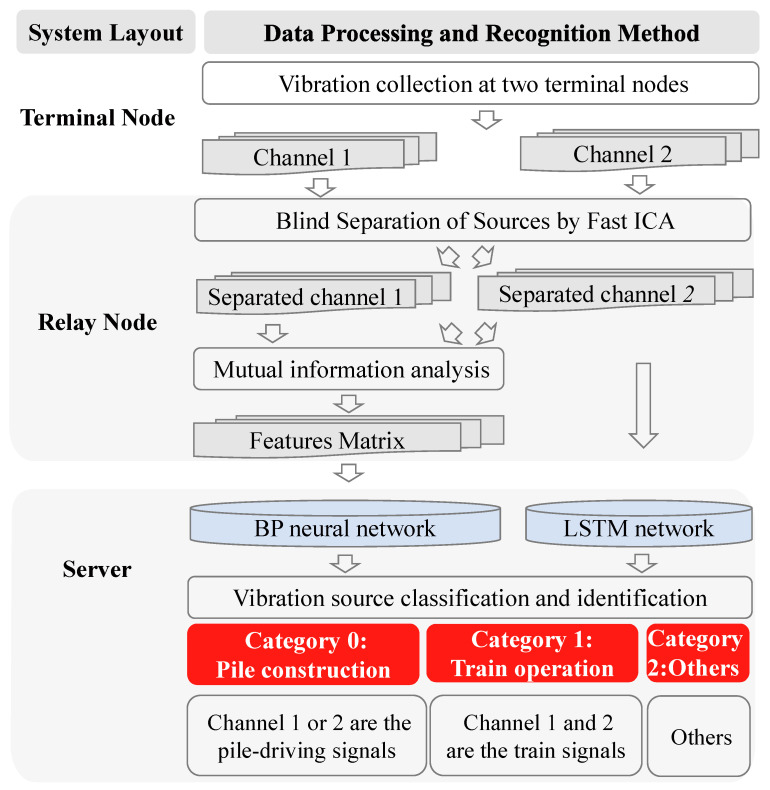
Flow chart describing the two evaluated methods for the intelligent recognition of nearby construction behavior.

Corresponding to the three levels of the monitoring system, an intelligent solution for data processing is proposed herein, as shown in Figure 2.

- Signal collection and sampling at terminal node

To meet the requirements of the signal acquisition for subsequent blind source separation, the number of sensors needs to be greater than the number of signal types to be identified, i.e., more than two. Here, by taking two single-channel sensors as an example, two channels of signals can be obtained at the terminal point, and the signals will be further sampled by time windows of 10 s. Then, if the peak value of the sampled signal exceeds the environment levels, the data will be transmitted to the relay point.

- Blind source separation and MI analysis at relay node

After the sampled signal enters the relay node, the blind source separation technology is first applied to separate it into two channel signals with a limited number of monitoring object sources, including pile-driving vibration, train-induced vibration, and others; it will minimize the number of required classification categories. The details are introduced in Section 2.2 and Section 3.2.

Meanwhile, to improve the robustness and training efficiency of the intelligent recognition algorithm, the MI is applied at the relay node to analyze the correlation between the vibration features and the recognition objects; features with high correlation are then used to form a feature matrix that is transmitted back to the server. This matrix is employed by the intelligent network to reduce the input matrix dimensions and improve the system robustness and efficiency.

- Vibration source classification and construction behavior intelligent recognition at server

Finally, the separated two channels signals are transmitted back to the server, and the intelligent algorithm is used to determine the vibration signal category. Category 1 is the train operation case, with both channels of data determined to be train-induced signals. The second category is the determination of whether any channel data is determined to be pile-driving signals; in this case, the early warning is announced at once. If any channel does not contain a train response and a pile-driving signal, this situation will be classified as “others”.

To highlight the advantages of combining the MI algorithm, the MLP network is used for feature and signal source recognition; this approach is here termed as the MI-MLP method. Most traditional methods adopt similar solution methodologies, where the separated signal is directly transmitted to the server. For a comparison of the proposed and existing methods, the commonly used long short-term memory (LSTM) method is employed to determine the vibration signal categories.

### 2.2. Vibration Signal Blind Source Separation

In the scenario investigated in this study, the main vibration types include train operation-induced vibration and pile foundation construction-induced vibration. Therefore, an independent component analysis (ICA) algorithm based on negative entropy is applied, as it can effectively separate relatively independent signals according to their sources [31]. As negative entropy can provide non-Gaussian measurements of random variables, it can realize improved computational efficiency and robustness. Therefore, the FastICA algorithm based on negative entropy was employed in this study, as follows [32]:

(1) $$J(y)=H(y_{Gauss})-H(y)$$

where *J*(*y*) represents the negative entropy of the random variable *y*, *H*(*y*) represents the entropy value of the random variable *y*, and *y_Gauss_* represents a random variable with the same variance as that of *y* that conforms to the Gaussian distribution. According to the definition of entropy, the solution of negative entropy must be able to calculate the probability density of random variables. In actual use, the mean negative entropy value can be approximately expressed as

(2) $$J(y)=\{E[G(y)]-E{\left[G(y_{Gauss})]\right\}}^{2}$$

where *G* is a non-quadratic function, generally defined by

(3) $$G_{1}(y)=\frac{1}{k}\log[\cosh(ky)],\;1\leq k\leq 2$$

(4) $$G_{2}(y)=-\exp(\frac{y^{2}}{2})$$

The goal of the FastICA algorithm is to determine the y=Wx value that maximizes Equation (2). This optimization employs the Newton iterative method using the following procedure:

1. Generate a random unmixing matrix $W_{0}$ that satisfies $\|W_{0}\|=1$;
2. Assume $W_{k+1}=E\left[zG\left(W_{k}{}^{T}z\right)\right]-E\left[G^{\prime }\left(W_{k}{}^{T}z\right)\right]W_{k}$ and iterate *k*;
3. Standardize and make $W_{k+1}=W_{k+1}/\|W_{k+1}\|$;
4. When the convergence condition is verified according to $\left|W_{k+1}-W\right|<\varepsilon$, end the cycle step, and end the algorithm after outputting the final unmixing matrix to obtain the solution of independent component y=Wx; otherwise, return to step 2 and continue with the iterations.

### 2.3. Mutual Information Analysis Method

The MI method has proven to be an effective tool to measure interdependence by calculating the shared information between two random variables. In the proposed method, it is used to select independent features to form the input matrix of the MLP neural network. According to the MI analysis theory, the MI value between two random variables *X* and *Y* is defined as

(5) $$I(X,Y)=\sum_{y_{i}\in Y}\sum_{x_{i}\in X_{j}}p(X_{j}=x_{i},Y=y_{i})\log_{2}(\frac{p(X_{j}=x_{i},Y=y_{i})}{p(X_{j}=x_{i})p(Y=y_{i})})$$

where $p\left(X_{j}=x_{i}\right)$ denotes the probability that $X_{j}$ is equal to $x_{i}$, $p\left(Y=y_{i}\right)$ denotes the probability that *Y* is equal to *y_i_*, and $p\left(X_{j}=x_{i},Y=y_{i}\right)$ indicates the probability of both variables simultaneously matching their counterparts.

### 2.4. Intelligent Recognition Method

#### 2.4.1. MLP Neural Network

**Figure 3:**
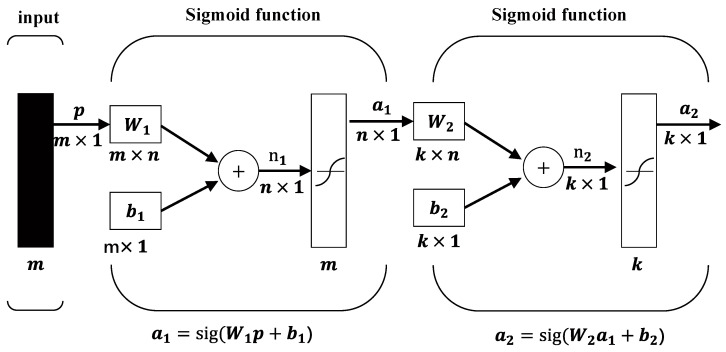
MLP neural network model structure.

Two intelligent signal source recognition methods were applied and compared in this study. With regard to the first method, as shown in Figure 3, the MLP recognition network used in this study is a fully connected MLP neural network employing error back propagation; it was constructed based on the TensorFlow module of Python. The sigmoid function is used as the activation function, and the cross-entropy function is used as the loss function. The input *p* of the network is an *m*-dimensional feature vector obtained through the MI. The weight *w_i_* in the hidden layer is an *m* × *n* matrix, while the bias *b_i_* is an *n*-dimensional feature vector.

#### 2.4.2. LSTM Method

**Figure 4:**
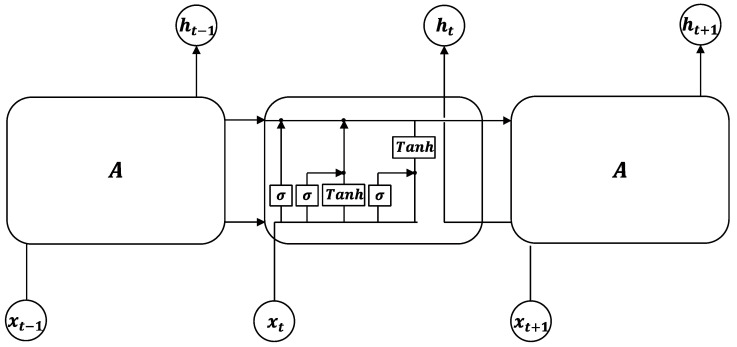
Internal structure of the LSTM computing layer.

The LSTM [33], illustrated in Figure 4, offers the advantage of being able to solve the gradient instability problem associated with recurrent neural networks, thus making it superior to the conventional MLP model. This is accomplished by adding a new cell *σ* and a gating system in memory module *A* [34]. Accordingly, the training of the LSTM employs the back propagation through time algorithm, which propagates the error term back along time and layers [35,36].

## 3. Vibration Signal Separation and Data Preparation

### 3.1. Vibration Measurement and Data Preparation

For obtaining the vibration signals required for the subsequent feature analysis and intelligent recognition, considering the difficulty in acquiring mixed vibration signals from an operational subway tunnel, field tests were only conducted at two sites in order to collect train-induced and pile-driving vibration signals.

#### 3.1.1. Pile-Driving-Induced Signals

One of the field tests was conducted in a power tunnel to collect the vibration signals induced by a nearby pile foundation construction. This tunnel was a pipe jacking tunnel with an outer diameter of 3.2 m and an inner diameter of 2.7 m. In the nearby construction, a steel pipe pile was being driven using the resonance hammer method. Six accelerometers were installed on the interior wall of three tunnel sections in order to collect the tunnel vibration responses in the radial direction at a sampling frequency of 1000 Hz. Overall, after being sampled with a time window of 10 s, 14,404 groups of pile-driving vibration signals with different distances from the power tunnel were obtained via this field test.

#### 3.1.2. Train-Operation-Induced Signals

Another field test was used to collect train-operation-induced vibrations; this test was conducted in Shanghai metro line 12, which is a shield tunnel with an outer diameter of 6.2 m and an inner diameter of 5.7 m. Eight accelerometers were arranged on the track bed and side walls of four different tunnel sections at intervals of 12 m. The accelerometers collected data at 1000 Hz for three months. Finally, the collected data were sampled using a time window of 10 s, and a total of 29,592 groups of signals were prepared for the following intelligent recognition.

#### 3.1.3. Others

For training and testing the classification of data other than pile-driving and train-vibration signals, the environment vibration data collected at the above two sites and other power and metro line tunnels were used, including the vibration response caused by ground traffic and human activities in the tunnel. Finally, 10,286 groups of signals were prepared.

### 3.2. Signal Separation Using FastICA and Comparison

As described in Section 2, the FastICA method was employed in this study to separate the mixed signals and thus minimize the number of classification categories. In this section, to verify the effect of separation, the mixed and single-type signals were prepared for separation. The mixed signals were obtained by combining the two types of signals with different weights.

#### 3.2.1. The Separation of Train Operation and Pile-Driving Mixed Signals

**Figure 5:**
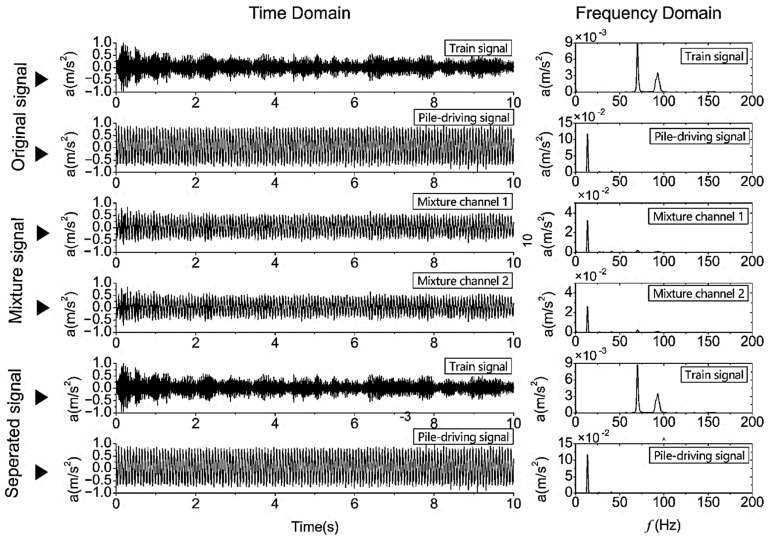
Example of mixed signals and separation using the FastICA method.

Since it is difficult to obtain the measured mixed signal, the pile-driving- and train-operation-induced signals were therefore combined with different weights to form two-channel mixed signals; the mixture and separation results are shown in Figure 5.

From Figure 5, two signals of train-operation-induced and pile-driving signals were combined by two random weights between 0.35 and 0.5, forming two signal mixtures, mixture channel 1 and mixture channel 2. Then, by using the FastICA-based separation algorithm, it found that only two separated signals can be obtained from the mixed signals.

As shown in Figure 5, before and after signal separation, the time domain waveform and frequency components are well preserved. It shows that the mixed signal will be well restored to the source signal, so as to reduce the number of classifications.

#### 3.2.2. The Separation of Single-Type Signals

**Figure 6:**
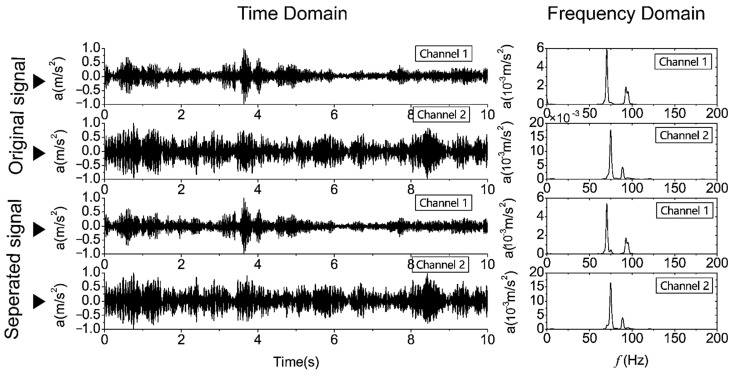
Example of two-channel single-type signal separation using the FastICA method.

More commonly, the input two-channel signals are of a single type, either the train-operation-induced or the pile-driving-induced signals. Because the time and frequency characteristics of train-operation-induced signals are more sophisticate than those of pile-driving signals, for illustrating the separation of a single-type signal, two channels of train-operation-induced signals will be used as an example. The results are shown in Figure 6. It was also found that the waveform and frequency components are well preserved before and after separation. This means that, for a single signal, blind source separation can retain the characteristics of the original signal.

**Figure 7:**
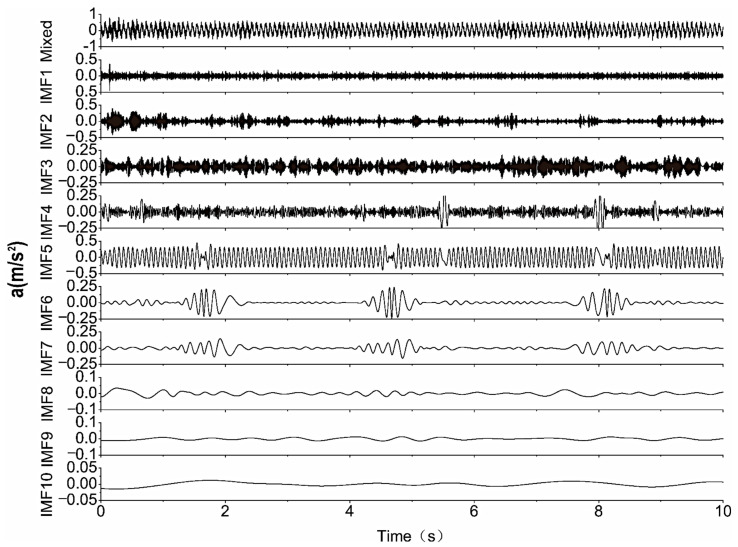
IMF components by performing EMD for the mixed signal.

For further comparison of the advantages of the blind source separation method, the HHT method was used [37]. The results in terms of performing empirical mode decomposition (EMD) on the mixed signal are presented in Figure 7; ten IMFs can be identified using EMD. Additionally, the time-domain characteristics are significantly different from those of the original signal prior to synthesis.

As shown above, the aim of signal separation is to limit the types of typical signals in order to reduce the number of intelligent recognition categories. Additionally, the blind source separation discussed above can separate signal sources from different collecting channels, not only for the mixed signals, but also for the single type signals, without creating any new signals.

### 3.3. Dataset Preparation and Features Analysis

#### 3.3.1. Sample Preparation and Dataset Construction

Based on the original signals collected at the field sites, three parts of the data were prepared for the construction of training and testing datasets, including the pile-driving signals, train operational signals, and others.

As shown in Table 1, 13,184 groups of pile-driving signals, 23,832 groups of train operational signals, and 8229 groups of environment signals collected from the field sites were used for the training dataset.

In total, 1220 groups of pile-driving signals and 5760 groups of train operational data were used to conduct the signal separation, and the re-separated data were used for the test set. Among them, 1220 groups of pile-driving data and train operational data were used to form the mixed signals, and 4540 groups of train operational data were employed as two-channel data to achieve single-type signal separation.

#### 3.3.2. Typical Time and Frequency Domain Features

In order to explore the time and frequency domain characteristic distribution of the above dataset, and as a basis of the following MI analysis, sixteen time and frequency domain features were selected and are listed in Table 2, where *x_p_* is the peak value of the vibration, *N* is the length of the signal sampling window, and *S* and *E* are the beginning and end of the sampling signal, respectively.

**Figure 8:**
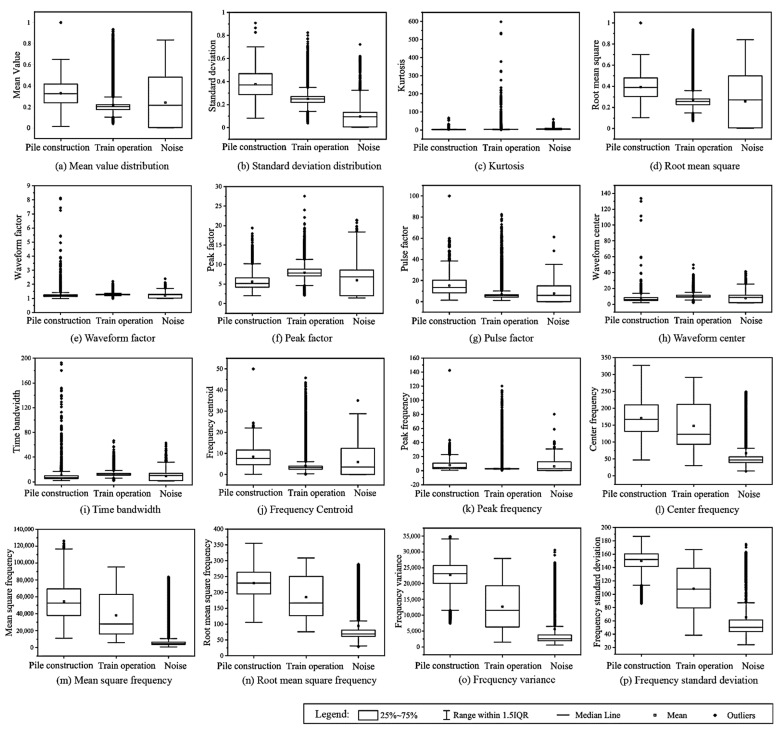
Statistical distribution of each feature on two types of target signal data.

Additionally, the box chart of the statistical distribution of each feature on three types of target signal data is shown in Figure 8.

As can be seen from Figure 8, for the pile-driving signals collected from sites at different distances from the sensor, and the train-induced vibration signals obtained at different times, the features show obvious discreteness, and have overlapping distribution areas, representing a challenge for the MLP network. Meanwhile, it was also found that the discreteness of the frequency-domain features is less than that of the time-domain features. Especially for the train operation signals, the feature distribution in the frequency domain is more stable than that in the time domain, and the increased use of the frequency features will increase the recognition accuracy.

## 4. Application of Combined MI-MLP Neural Network Method

In this section, the intelligent recognition of vibration signal sources using the combined MI-MLP neural network is discussed based on the data prepared, as described in Section 3. This process mainly consists of the MI analysis of vibration features, the feature selection and input matrix formation, and the training and testing of the MLP neural network.

### 4.1. MI Analysis and Feature Selection

To measure the correlation between the feature and the corresponding vibration category, the variable *X* can be treated as the feature vector and *Y* can be considered as the corresponding vibration-type label. When using *X_n_* groups of training and validation set samples, as listed in Table 1, there exists a feature vector *X_j_* with the dimensions *X_n_* × 1. A corresponding label vector representing the vibration types is assembled by assigning a category label to each row of the feature vector, namely $Y=\left\{y_{1},y_{2},y_{3},\ldots y_{i},\ldots ,y_{Xn}\right\}$, where $y_{i}$ is 0 when *x_i_* is the pile-construction-induced response and 1 when *x_i_* is the train-operation-induced response.

**Figure 9:**
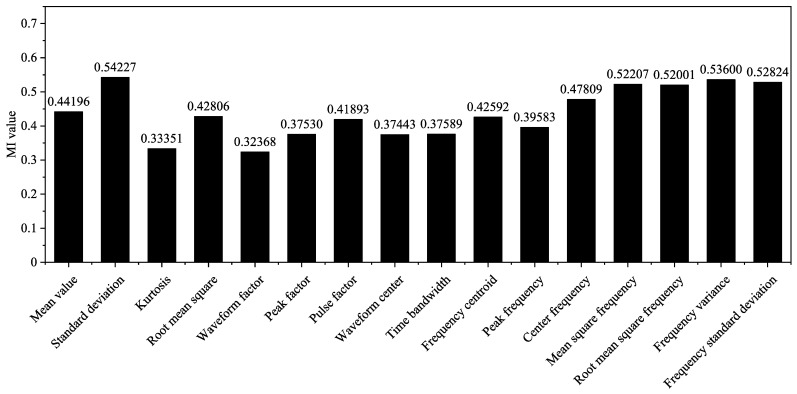
MI values between different features and the corresponding signal types.

The MI values of the sixteen feature vectors and the corresponding label vector are shown in Figure 9; from the figure, it is evident that the frequency domain features are most relevant to the vibration signal classification. Finally, the MI values of ten features were found to be greater than 0.4 and were accordingly selected to form the input matrix of the MLP neural network.

### 4.2. Training and Testing of the MLP Neural Network

Based on the method described in Section 5.1, 600 feature vectors were used as the input for the MLP network, instead of the 600 groups of vibration signals. The training set and test set were then constructed by randomly selecting values from these vectors at a ratio of 8:2.

Hyperparameter searching was performed when preparing the data set, as the selection of appropriate hyperparameters can ensure a smooth training process and improve the final model training quality. The most widely used hyperparameter automatic searching algorithms include the grid search method and the random search method. In the grid search algorithm, the specified parameter array grows from different sequences and then enters the training model to finally select the optimal set of parameters according to their accuracies. This approach is more suitable for cases involving fewer parameters; as the number of hyperparameters increases, the computational complexity of the grid search method increases exponentially. In contrast to the grid search method, the random search method does not need to go through all possible combinations of hyperparameters; instead, this approach defines their distributions. As a result, it has a lower calculation complexity and, consequently, a greater efficiency when searching for a large number of hyperparameters. Accordingly, this approach was employed in this study.

When using the random search method, the search routes begin from a random starting parameter combination and converge to a final parameter value according to the probability distribution of each parameter. For this purpose, in this study, a function was coded in Python that considered the hyperparameter distributions to be uniform, and the ratio of model accuracy to training time was used as the evaluation index. The search parameters and results are shown in Table 3.

The MLP neural network shown in Figure 3 was trained and tested using the ten feature vectors selected using MI analysis, as discussed in Section 4.1. Additionally, because of the time and frequency domain features used, there is a large gap between the two types of features in terms of values. Therefore, normalization was employed to the feature matrix before inputting it into the training and testing network.

**Figure 10:**
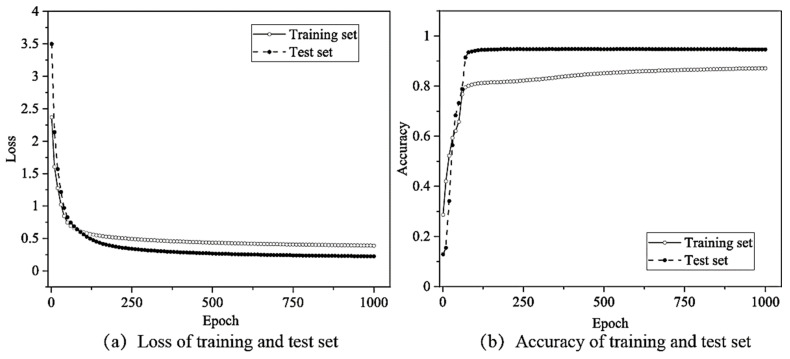
Loss function and accuracy changes according to epoch when using the MLP neural network.

The changes in the loss function with the number of MLP network iterations using the hyperparameter search results (Table 3) are shown in Figure 10a. Clearly, the loss function decreased rapidly over the first fifty epochs before converging to 0.39 in the training set and 0.23 in the test set. Additionally, from Figure 10b, it can be observed that the final accuracy of the network structure when using the training set was near 87.17%, and near 94.68% when using the test set.

### 4.3. The Effect of MI Analysis and Feature Selection

**Figure 11:**
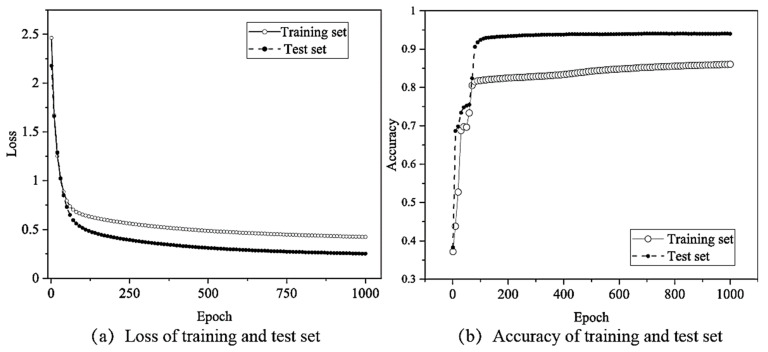
Loss and accuracy changes by MLP neural network without feature selection.

The feature matrix after MI analysis and the original signal samples were separately used as the input of the MLP model to evaluate the effects of feature selection on the loss and accuracy of the training and validation sets (Figure 11).

**Figure 12:**
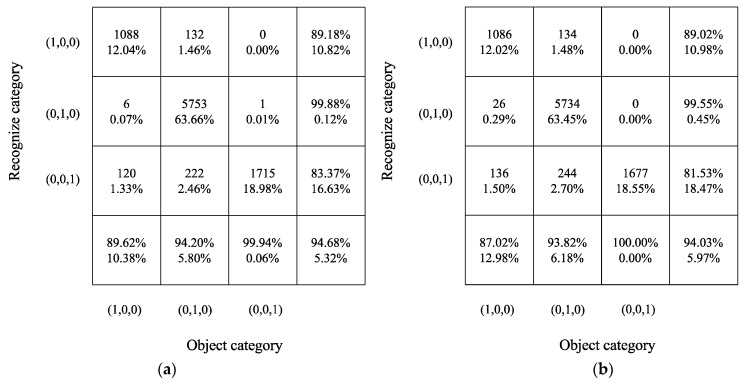
Confusion matrix for vibration source recognition by the MLP neural network without feature selection. (**a**) MI + MLP; (**b**) MLP.

The change in the loss function and recognition accuracy during model training, obtained when using the MI analysis and feature selection, are shown in Figure 9. Additionally, the training results without feature selection are shown in Figure 12 (all the 16 features were used). The training loss converged slowly, only reaching convergence after 1000 epochs; the resulting accuracy was only 86.05%. Similarly, the loss of the test set converged after nearly 1000 epochs, and the accuracy only reached 94.03%, decreasing by 0.65% before feature selection. Thus, the use of feature selection can improve the training speed and recognition accuracy of the vibration signal source recognition method.

Additionally, as shown in Figure 12, the confusion matrix also shows that the recognition accuracy of all the categories will decrease if the MI and feature selection are not used, and the total accuracy decreases by 0.65%. Therefore, feature selection by the MI method can improve the recognition accuracy.

## 5. Comparison with LSTM Network-Based Recognition Method

### 5.1. Hyperparameter Searching

Hyperparameter searching was then performed using the random search method, similar to that performed for the MI-MLP method, as described in Section 4; the results are shown in Table 4.

### 5.2. Training and Testing of LSTM Network

**Figure 13:**
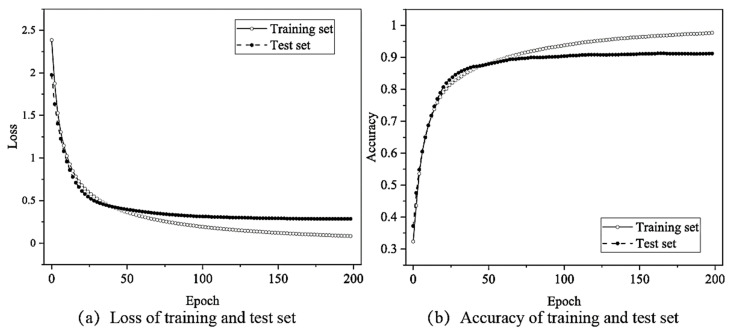
Change in the loss function and the accuracy of the training and validation sets according to epoch when using the LSTM network.

The LSTM network was applied to identify the vibration signals, as described in Section 2.4 and shown in Figure 3. It should be noted that because the LSTM network offers advantages in identifying discrete time series, the training and validation signal samples were divided into ten time steps and directly used as the network input (Figure 13).

**Figure 14:**
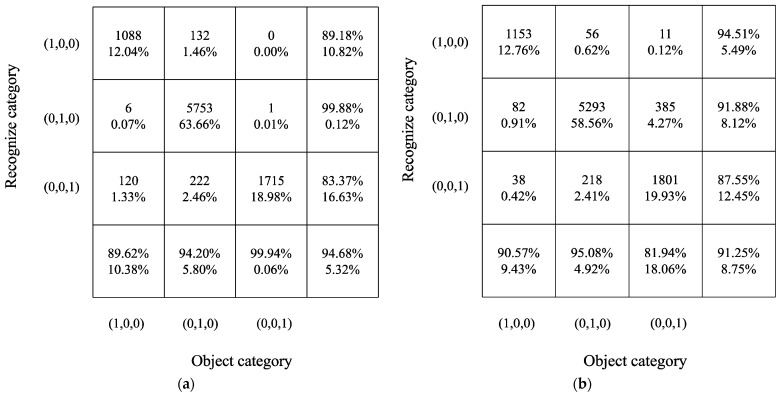
Confusion matrix for vibration source recognition by the MLP neural network and LSTM. (**a**) MI-MLP method; (**b**) LSTM.

Based on the hyperparameter search results, as listed in Table 4, the changes in the loss function with the number of iteration epochs were determined, as shown in Figure 14a. It can be observed that the loss function decreases rapidly over the first twenty epochs, before finally converging to 0.08 for the training set and approximately 0.28 for the test set. From Figure 7, it can be observed that the final accuracy of the network structure over the training set was 97.7% and 91.25% over the test set.

### 5.3. Method Comparison

Meanwhile, the details of the recognition results are shown in the confusion matrix in Figure 14. The object category (1, 0, 0) (pile-driving-induced vibration recognition) achieved an accuracy of 94.51%, correctly identifying 54 of the 75 signals, higher than that achieved by the MLP + MI method; object category (0, 1, 0) (train operation-induced vibration recognition) achieved an accuracy of 91.88%, correctly identifying 5293 of the 5760 signals; and the total accuracy of the LSTM-based vibration signal recognition was 91.25%, which is lower than the recognition accuracy obtained when using the MI-MLP method.

The LSTM has been proven to be a particularly efficient network and good at time series prediction; therefore, the recognition accuracy for pile-driving and environment vibration is higher than that of the MLP network. However, for the train operational signals, as shown in Figure 8, the frequency-domain feature distribution concentration is much better than that of the time-domain feature, and future compression and selection by the MI method greatly improve the recognition accuracy. Meanwhile, the training of the LSTM network took 525.35 s, much longer than that of the MLP network, which was 20.02 s. This indicates that the use of suitable feature selection can considerably improve the training efficiency of vibration source recognition.

## 6. Conclusions

This paper proposed a method for the real-time monitoring and early warning of surrounding construction behaviors near underground facilities. Considering the challenges associated with intelligent recognition owing to vibration mixing and signal acquisition difficulties, the MI analysis and blind source separation methods were employed. The conclusions of this work can be detailed as follows:

- The recognition accuracies obtained by the LSTM and MI-MLP methods when using the test set were both greater than 90%, which can meet the needs of practical applications. This demonstrates the feasibility of real-time construction monitoring with suitable recognition robustness.
- Blind source separation can decompose mixed signals into a limited number of identifiable samples, which can help minimize the number of classification categories caused by signal mixing and improve recognition accuracy.
- Feature compression and selection after MI analysis can increase the training efficiency and recognition accuracy. Especially for signals with a centralized distribution of frequency domain features, the recognition accuracy of the MLP network will have a better performance than that of a well-known time series prediction LSTM model.

The results of this study can be used to develop a real-time vibration monitoring system for underground infrastructure that is capable of identifying the construction activity source of a particular vibration. This will help ensure the safety of underground infrastructure, such as transit and pipeline networks, near construction sites. Although the proposed method was evaluated in this study using pile-driving vibrations, it should be capable of identifying a wide range of construction activities. Therefore, future research should focus on applying the proposed method to identify various construction activities based on measured vibrations.

## Figures and Tables

**Table 1:** Division of sample sets for intelligent model.

	Training Set (Group)	Test Set (Group)
Pile-driving signals	13,184 (original signal)	1220 (separation signal)
Train operational signals	23,832 (original signal)	5760 (separation signal)
Others (noise, etc.)	8229 (original signal)	2057 (original signal)

**Table 2:** Selected features for MI analysis and feature compression.

Feature	Equation	Feature	Equation
Mean value	$\mu_{x}\left(t\right)=\frac{1}{N}\overset{E}{\underset{i=S}{\sum }}x_{i}=E\left(X\right)$	Kurtosis factor	$KF=\frac{\sum_{i=S}^{E}x_{i}^{4}}{\sqrt{\sum_{i=S}^{E}x_{i}^{2}}}$
Standard deviation	$\sigma_{x}=\sqrt{\frac{1}{N}\overset{E}{\underset{i=s}{\sum }}{\left(x_{i}-\mu_{x}\right)}^{2}}$	Pulse factor	$C_{pul}=\frac{x_{p}}{\mu_{x}}$
Kurtosis	$K=\frac{1}{N}\overset{E}{\underset{i=S}{\sum }}{\left(\frac{x_{i}-\mu_{x}}{\sigma_{x}}\right)}^{4}$	Clearance factor	$C_{clea}=\frac{x_{p}}{{\left(\frac{1}{N}\sqrt{\left|x_{i}\right|}\right)}^{2}}$
Root mean square	$x_{rms}=\sqrt{\frac{x_{S}{}^{2}+x_{S+1}{}^{2}+\ldots +x_{E}{}^{2}}{E-S+1}}$	Waveform center	$\langle t\rangle =\frac{1}{N}\overset{E}{\underset{i=S}{\sum }}(i\cdot |x_{i}{|}^{2})$
Wave form factor	$C_{Ws}=\frac{x_{rms}}{\mu_{x}}$	Time width	$\sigma_{t}=\sqrt{\overset{E}{\underset{i=S}{\sum }}{\left(i-\langle t\rangle \right)}^{2}{\left|x_{i}\right|}^{2}}$
Peak factor	$C_{f}=\frac{x_{p}}{x_{rms}}$	Mean square frequency	$MSF=\frac{\int_{0}^{\infty }f^{2}FFT\left(x,t\right)df}{\int_{0}^{\infty }FFT\left(x,t\right)df}$
Center frequency	$FC=\frac{\int_{0}^{\infty }fFFT\left(x,t\right)df}{\int_{0}^{\infty }FFT\left(x,t\right)df}$	Root mean square frequency	$RMSF=\sqrt{MSF}$
Frequency variance	$VF=\frac{\int_{0}^{\infty }{\left(f-FC\right)}^{2}FFT\left(x,t\right)df}{\int_{0}^{\infty }FFT\left(x,t\right)df}$	Frequency standard deviation	$RVF=\sqrt{VF}$

**Table 3:** Setting and results of hyperparameter searching for the MLP neural network.

Search Parameters			
Search range of learning rate	[0, 1)	Iteration step for learning rate	0.01
Search range of calculation layers	[1, 3)	Number of calculation layers in each search step	1
Search range of hidden layer nodes	[1, 100)	Number of hidden layer nodes in each search step	10
Search results			
Optimal learning rate	0.1	Optimal number of hidden layer nodes	10
Optimal number of computing layers	2	Optimal accuracy	94.68%

**Table 4:** Setting and results of LSTM network hyperparameter searching.

Search Parameters			
Search range of learning rate	[0, 1)	Iteration step for learning rate	0.01
Search range of calculation layers	[1, 3)	Number of calculation layers in each search step	1
Search range of hidden layer nodes	[1, 100)	Number of hidden layer nodes in each search step	10
Search results			
Optimal number of computing layers	2	Optimal accuracy	91.25%
Optimal number of hidden layer nodes	50		

## Data Availability

The data presented in this study are available on request from the corresponding author.
